# Supplementary material for: Prefrontal cortex supports speech perception in listeners with cochlear implants
Source: eLife. 2022 Jun 6;11:e75323. doi: 10.7554/eLife.75323 (PMC9225001; doi:10.7554/eLife.75323)
Supplement: Figure 3—source data 1. [file elife-75323-fig3-data1.docx]

**Figure 3—source data1 |** Noisy source-detector numbers are provided along with the threshold used for identifying them for each CI user.

| **Subject ID** | **Noisy Source #** | **Noisy Detector #** | **Threshold (% max)** |
| --- | --- | --- | --- |
| DOTCI01 | 69, 74 | 33, 65, 66, 70 | 30 |
| DOTCI02 | - | 66 | 30 |
| DOTCI03 | 69, 70 | 70 | 50 |
| DOTCI04 | - | 70 | 30 |
| DOTCI05 | 12, 69, 77 | 70, 74 | 30 |
| DOTCI06 | 66 | 61, 62 | 30 |
| DOTCI07 | 78 | - | 30 |
| DOTCI08 | 69 | 67, 70 | 30 |
| DOTCI10 | 70 | 66 | 30 |
| DOTCI11 | 8, 70, 74 | 71 | 30 |
| DOTCI12 | 79 | 77, 80 | 30 |
| DOTCI13 | - | 69 | 30 |
| DOTCI14 | 74 | - | 60 |
| DOTCI15 | - | 33 | 50 |
| DOTCI16 | 65 | 68 | 50 |
| DOTCI17 | 73 | 70 | 40 |
| DOTCI18 | - | 66, 73 | 50 |
| DOTCI19 | 82 | 78, 79 | 70 |
| DOTCI20 | - | 66, 70 | 50 |
| DOTCI21 | 65 | 61 | 80 |
