# Supplementary material for: Prefrontal cortex supports speech perception in listeners with cochlear implants
Source: eLife. 2022 Jun 6;11:e75323. doi: 10.7554/eLife.75323 (PMC9225001; doi:10.7554/eLife.75323)
Supplement: Transparent reporting form [file elife-75323-transrepform1.docx]

***eLife’s* transparent reporting form**

We encourage authors to provide detailed information *within their submission* to facilitate the interpretation and replication of experiments. Authors can upload supporting documentation to indicate the use of appropriate reporting guidelines for health-related research (see [EQUATOR Network](http://www.equator-network.org/%20)), life science research (see the [BioSharing Information Resource](https://biosharing.org/)), or the [ARRIVE guidelines](http://www.plosbiology.org/article/info:doi/10.1371/journal.pbio.1000412) for reporting work involving animal research. Where applicable, authors should refer to any relevant reporting standards documents in this form.

If you have any questions, please consult our Journal Policies and/or contact us: [editorial@elifesciences.org](mailto:editorial@elifesciences.org).

**Sample-size estimation**

- You should state whether an appropriate sample size was computed when the study was being designed
- You should state the statistical method of sample size computation and any required assumptions
- If no explicit power analysis was used, you should describe how you decided what sample (replicate) size (number) to use

Please outline where this information can be found within the submission (e.g., sections or figure legends), or explain why this information doesn’t apply to your submission:

We explained our rationale for sample size and effect size in “Quantification and statistical analysis” section.

**Replicates**

- You should report how often each experiment was performed
- You should include a definition of biological versus technical replication
- The data obtained should be provided and sufficient information should be provided to indicate the number of independent biological and/or technical replicates
- If you encountered any outliers, you should describe how these were handled
- Criteria for exclusion/inclusion of data should be clearly stated
- High-throughput sequence data should be uploaded before submission, with a private link for reviewers provided (these are available from both GEO and ArrayExpress)

We provided a clear exclusion/inclusion criterion for excluding 2 subjects based on low pulse SNR and high motion, which we elaborated in Appendix 1.

Please outline where this information can be found within the submission (e.g., sections or figure legends), or explain why this information doesn’t apply to your submission:

**Statistical reporting**

- Statistical analysis methods should be described and justified
- Raw data should be presented in figures whenever informative to do so (typically when N per group is less than 10)
- For each experiment, you should identify the statistical tests used, exact values of N, definitions of center, methods of multiple test correction, and dispersion and precision measures (e.g., mean, median, SD, SEM, confidence intervals; and, for the major substantive results, a measure of effect size (e.g., Pearson's r, Cohen's d)
- Report exact p-values wherever possible alongside the summary statistics and 95% confidence intervals. These should be reported for all key questions and not only when the p-value is less than 0.05.

Please outline where this information can be found within the submission (e.g., sections or figure legends), or explain why this information doesn’t apply to your submission:

Our main conclusion, the involvement of the left prefrontal cortex in speech perception in listeners with cochlear implants, has been based on the one-tailed two-sample t-test across N = 18 controls and N = 20 CI users and 95% confidence interval criteria (Figure 5). The p-value is reported in the figure caption and page 10 (p = 0.015) which is less than p = 0.016 corrected for multiple comparisons.

Our second important statistical significance is the decrease in the activity of the right auditory cortex in CI users during speech perception. This conclusion is also based on the two-tailed two-sample t-test across N = 18 controls and N = 20 CI users and 95% confidence interval criteria (Figure 5). The p-value is reported in the figure caption and page 11 (p = 0.0017) which is less than p = 0.016 corrected for multiple comparisons.

In figure 3 (multi-session single subject data), we used the t-map threshold of t = 3.1 which is equivalent of p < 0.01 for the one sample t-test of N = 12 spoken word recognition runs (B) and N = 4 spatial working memory runs (C). However, we are not making any statistical conclusions based on this figure.

In figure 2A, we used the t-map threshold of t = 1.6 which is equivalent of p < 0.05 for the one sample t-test of N = 9 spatial working memory sessions. We used this map to define an independent region of interest and we are not making any statistical conclusions based on this figure.

In figure 4, we used the t-map threshold of t = 1.6 which is equivalent of p < 0.05 for one sample t-tests of N = 18 for controls and N = 20 for CI users. To overcome the multiple comparisons problem, we perform ROI-based analysis for 3 ROIs in Figure 5.

In Figure 6, we interpreted the results based on the 95% confidence interval criteria (p = 0.05) threshold. We found that the activity in dorsolateral prefrontal cortex in CI users during speech processing is correlated with unaided left ear pure tone average thresholds, with statistical significance of p = 0.01. As mentioned in the “Behavioral measures” section, these were exploratory analysis and not part of the main hypothesis of our study. For each plot, both p-values and Pearson r values are also shown in the plot and in the associated text in the paper.

All data used in these figures, as well as the scripts used for performing these statistical analyses are shared with figure numbers as file and script names in the public OSF repository (<https://osf.io/nkb5v/?view_only=2c8ef3af126542a49be055d50ac935d4>).

(For large datasets, or papers with a very large number of statistical tests, you may upload a single table file with tests, Ns, etc., with reference to sections in the manuscript.)

**Group allocation**

- Indicate how samples were allocated into experimental groups (in the case of clinical studies, please specify allocation to treatment method); if randomization was used, please also state if restricted randomization was applied
- Indicate if masking was used during group allocation, data collection and/or data analysis

Please outline where this information can be found within the submission (e.g., sections or figure legends), or explain why this information doesn’t apply to your submission:

We had two distinct samples. 1. Right ear cochlear implant users. 2. Normal hearing controls.

All behavioral measures for these two groups are presented in Table 1. We also shared individual measures of these scores in 2 csv file called “auditory_thresholds” and “cidot_brain_behavior” in the OSF repository.

**Additional data files (“source data”)**

- We encourage you to upload relevant additional data files, such as numerical data that are represented as a graph in a figure, or as a summary table
- Where provided, these should be in the most useful format, and they can be uploaded as “Source data” files linked to a main figure or table
- Include model definition files including the full list of parameters used
- Include code used for data analysis (e.g., R, MatLab)
- Avoid stating that data files are “available upon request”

Please indicate the figures or tables for which source data files have been provided:

All numerical data and source code (both MATLAB scripts and SPM parameters) for producing all maps and plots for all main figures and most figure supplements are provided in the OSF repository with instructions for the order of analysis.
